# Cardiovascular/Stroke Risk Assessment in Patients with Erectile Dysfunction—A Role of Carotid Wall Arterial Imaging and Plaque Tissue Characterization Using Artificial Intelligence Paradigm: A Narrative Review

**DOI:** 10.3390/diagnostics12051249

**Published:** 2022-05-17

**Authors:** Narendra N. Khanna, Mahesh Maindarkar, Ajit Saxena, Puneet Ahluwalia, Sudip Paul, Saurabh K. Srivastava, Elisa Cuadrado-Godia, Aditya Sharma, Tomaz Omerzu, Luca Saba, Sophie Mavrogeni, Monika Turk, John R. Laird, George D. Kitas, Mostafa Fatemi, Al Baha Barqawi, Martin Miner, Inder M. Singh, Amer Johri, Mannudeep M. Kalra, Vikas Agarwal, Kosmas I. Paraskevas, Jagjit S. Teji, Mostafa M. Fouda, Gyan Pareek, Jasjit S. Suri

**Affiliations:** 1 Department of Cardiology, Indraprastha APOLLO Hospitals, New Delhi 110076, India; drnnkhanna@gmail.com; 2 Department of Biomedical Engineering, North Eastern Hill University, Shillong 793022, India; mahesh.nehu.333@gmail.com (M.M.); sudip.paul.bhu@gmail.com (S.P.); 3 Stroke Monitoring and Diagnostic Division, AtheroPoint^TM^, Roseville, CA 95661, USA; drindersingh1@gmail.com; 4 Department of Urology, Indraprastha APOLLO Hospitals, New Delhi 110076, India; ajitsaxena@hotmail.com; 5 Max Institute of Cancer Care, Max Super Specialty Hospital, New Delhi 110017, India; puneet1923@gmail.com; 6 College of Computing Sciences and IT, Teerthanker Mahaveer University, Moradabad 244001, India; phd.jiit@gmail.com; 7 Department of Neurology, Hospital del Mar Medical Research Institute, 08003 Barcelona, Spain; elisacua@gmail.com; 8 Division of Cardiovascular Medicine, University of Virginia, Charlottesville, VA 22908, USA; as8ah@hscmail.mcc.virginia.edu; 9 Department of Neurology, University Medical Centre Maribor, 2000 Maribor, Slovenia; omerzu.tomaz@gmail.com (T.O.); monika.turk84@gmail.com (M.T.); 10 Department of Radiology, University of Cagliari, 09124 Cagliari, Italy; lucasabamd@gmail.com; 11 Cardiology Clinic, Onassis Cardiac Surgery Centre, 176 74 Athens, Greece; sophie.mavrogeni@gmail.com; 12 Heart and Vascular Institute, Adventist Health St. Helena, St. Helena, CA 94574, USA; lairdjr@ah.org; 13 Academic Affairs, Dudley Group NHS Foundation Trust, Dudley DY1 2HQ, UK; george.kitas@gmail.com; 14 Arthritis Research UK Epidemiology Unit, Manchester University, Manchester M13 9PL, UK; 15 Department of Physiology & Biomedical Engineering, Mayo Clinic College of Medicine and Science, Rochester, NY 55905, USA; fatemi.mostafa@mayo.edu; 16 Division of Urology, Department of Surgery, University of Colorado Anschutz Medical Campus, Aurora, CO 80045, USA; al.barqawi@cuanschutz.edu; 17 Men’s Health Centre, Miriam Hospital Providence, Providence, RI 02906, USA; martin_miner@brown.edu; 18 Department of Medicine, Division of Cardiology, Queen’s University, Kingston, ON K7L 3N6, Canada; amerschedule@gmail.com; 19 Department of Radiology, Harvard Medical School, Boston, MA 02115, USA; mkalra@mgh.harvard.edu; 20 Sanjay Gandhi Postgraduate Institute of Medical Sciences, Lucknow 226014, India; vikasagr@yahoo.com; 21 Department of Vascular Surgery, Central Clinic of Athens, 106 80 Athens, Greece; paraskevask@hotmail.com; 22 Ann and Robert H. Lurie Children’s Hospital of Chicago, Chicago, IL 60611, USA; jteji@mercy-chicago.org; 23 Department of Electrical and Computer Engineering, Idaho State University, Pocatello, ID 83209, USA; mfouda@ieee.org; 24 Minimally Invasive Urology Institute, Brown University, Providence, RI 02912, USA; gyan_pareek@brown.edu

**Keywords:** erectile dysfunction, pathophysiology, atherosclerosis, cardiovascular disease, carotid artery disease, carotid ultrasound-based tissue characterization, machine learning, deep learning, risk assessment

## Abstract

Purpose: The role of erectile dysfunction (ED) has recently shown an association with the risk of stroke and coronary heart disease (CHD) via the atherosclerotic pathway. Cardiovascular disease (CVD)/stroke risk has been widely understood with the help of carotid artery disease (CTAD), a surrogate biomarker for CHD. The proposed study emphasizes artificial intelligence-based frameworks such as machine learning (ML) and deep learning (DL) that can accurately predict the severity of CVD/stroke risk using carotid wall arterial imaging in ED patients. Methods: Using the PRISMA model, 231 of the best studies were selected. The proposed study mainly consists of two components: (i) the pathophysiology of ED and its link with coronary artery disease (COAD) and CHD in the ED framework and (ii) the ultrasonic-image morphological changes in the carotid arterial walls by quantifying the wall parameters and the characterization of the wall tissue by adapting the ML/DL-based methods, both for the prediction of the severity of CVD risk. The proposed study analyzes the hypothesis that ML/DL can lead to an accurate and early diagnosis of the CVD/stroke risk in ED patients. Our finding suggests that the routine ED patient practice can be amended for ML/DL-based CVD/stroke risk assessment using carotid wall arterial imaging leading to fast, reliable, and accurate CVD/stroke risk stratification. Summary: We conclude that ML and DL methods are very powerful tools for the characterization of CVD/stroke in patients with varying ED conditions. We anticipate a rapid growth of these tools for early and better CVD/stroke risk management in ED patients.

## 1. Introduction

Erectile dysfunction (ED) is a multi-factorial illness that is characterized by the presence of vascular atherosclerosis and hormonal, lifestyle, age, neurological, and physiological factors, all occurring in a well-coordinated manner [1,2]. Among all of the listed characteristics, vascular disease is the most common cause of ED [3]. Testosterone levels, psychological concerns, such as performance anxiety, and iatrogenesis are all the variables that contribute to ED development [4,5]. According to a variety of demographic studies, ED affects up to 150 million men globally [6,7]. As the world’s population ages, the prevalence of ED is expected to climb to 300 million men by 2025 [8,9]. Males aged 18–75 years in Europe had a prevalence of 19%, but men in the same age range in the UK had a prevalence of 39% for life ED and 26% for current ED [8,10,11].

ED has been linked to future cardiovascular events (CVE) in various studies [12,13], showing a high mortality rate due to CVD and stroke. Various studies have shown that ED patients had a considerably higher CVD risk than non-ED patients [14,15,16]. The most prominent risk factors associated with ED and CVD are diabetes, dyslipidemia, hypertension, smoking, and obesity, which lead to the development of oxidative stress, the primary cause of endothelial dysfunction [11,17]. Due to the reduction in endothelium-dependent vasodilation, there have been changes in structural vascular abnormalities, such as increased carotid intima-media thickness (cIMT) and the formation of atherosclerotic plaques [18,19,20].

**Figure 1:**
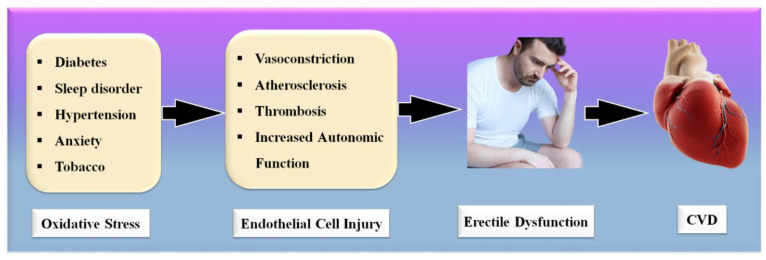
Relationship between CVD risk factors, ED, and CVD.

Significantly, the majority of male sexual ED is now recognized to be arterial in origin, with endothelial dysfunction serving as the common link [21,22]. The patient and his spouse are both negatively affected by ED, stressing the need for addressing ED as soon as feasible [23]. Figure 1 indicates the relationship between CVD risk factors and ED. From the above, we conclude that “There is a clear correlation between ED and CVD.” A comprehensive investigation of ED and CVD can be beneficial in the early diagnosis of heart attacks, strokes, and other unfavorable CVE [24,25].

Several changes occur as a result of the advancement of ED, including the creation of exudates, bleeding, and other symptoms [26]. These modifications have been implicated in the development of CVD [16]. Patients in the more severe phases of ED have a higher risk of CVD, and once a patient has been diagnosed with a CVD risk, coronary imaging is indicated to stratify the risks [13]. Also essential for visualizing the plaque in COAD, coronary artery imaging (CAI) is vital [27]. Intravascular ultrasonography and coronary angiography are the most frequently used imaging modalities for the visualization of coronary plaque [28,29].

The imaging modalities are costly and difficult to get one’s hands on, especially in underdeveloped nations [30]. As a result, it seems sensible to explore low-cost alternative imaging technologies that can still monitor CTAD in ED patients and risk-stratify them [20,31]. Vascular imaging technologies are useful for the treatment and can save lives before they become life-threatening [19]. Because the carotid artery and the coronary artery have genetically similar compositions, B-mode carotid ultrasonography is a preferred alternative for CTAD imaging of the carotid artery [32]. Image-based phenotypes such as carotid intima-media thickness and carotid total plaque area can be used as CVD surrogates. Further, accurate and automated carotid plaque burden quantification, risk stratification, and early monitoring of atherosclerotic disease in ED patients is therefore required [33].

Artificial intelligence (AI)-based methods have recently played a vital role in computer-aided diagnosis [34,35], especially in the detection and classification of several diseases [36,37]. Machine learning applications in medical imaging have just lately risen to prominence, such as diabetes [38]; the risk stratification of cancer types such as thyroid [39], liver [37,40], prostate [41,42], and ovarian [43]; vascular screening [44]; coronary artery disease risk characterization [45,46]; and surrogate biomarker CTAD imaging and its risk stratification [47,48]. Previously, ML models were developed to predict CVD, as it contains a variety of features from the CVD datasets [49,50,51]. Recently, the DL algorithms have been used to segment the carotid plaque wall thickness [52,53] for CVD risk assessment. As a result, it may be conceivable to use these AI-based solutions to handle CVD and stroke risk stratification in ED patients.

The objective of the proposed review study is to understand (a) the clinical linking between ED and CVD and vice versa, along with the risk factors of CVD in ED patients, and (b) the CVD risk stratification for the severity of heart failure and stroke in ED patients based on AI. One can use the risk factors such as office-based biomarkers (OBBM), laboratory-based biomarkers (LBBM), carotid ultrasound image phenotypes (CUSIP), and medicine usage (MedUSE) combined with ED covariates for designing knowledge-based systems for CVD prediction. Thus, ML and DL solutions can help in establishing the early CVD risk assessment of ED patients who are at a high risk of CVD or ischemic and hemorrhage stroke.

The following is an outline for the proposed review. Section 2 presents the PRISMA model for selecting ED-CVD-based studies. Section 3 presents the evidence of a link between ED and CVD based on the clinical evidence of shared risk factors, while Section 4 explains the biological link between ED and CVD. An AI-based system for a CVD/stroke risk assessment for ED patients is presented in Section. Section 4 presents the recommendations, manifestation, and treatment of ED. A critical 5discussion is presented in Section 5, leading to conclusions in Section 6.

## 2. Search Strategy

**Figure 2:**
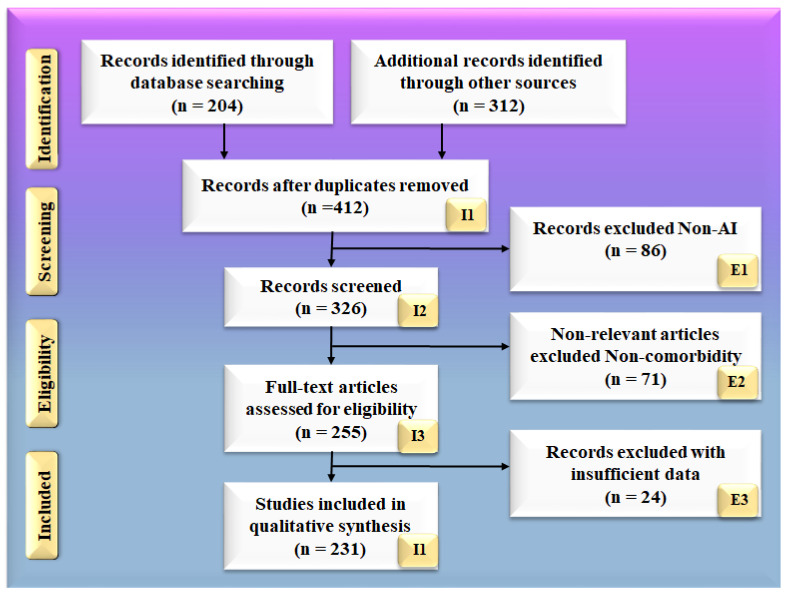
PRISMA model for selection of studies.

The search approach was based on the PRISMA paradigm, as shown in Figure 2. PubMed and Google Scholar are two major databases that were used to identify and screen relevant papers using keywords such as “cardiovascular disease”, “stroke”, “ED”, “Stroke and CVD”, “Erectile Dysfunction and CVD”, “Erectile Dysfunction and Stroke”, “carotid imaging”, “Erectile Dysfunction and artificial intelligence”, “atherosclerotic tissue classification and characterization in Erectile dysfunction”, “artificial intelligence”, and “Erectile Dysfunction and artificial intelligence”. When searching through the mentioned databases, a total of 204 entries were initially discovered. Furthermore, 312 entries were discovered through additional sources. Following the use of quality custom criteria such as time and relevance, this was reduced to 412 articles. A total of 326 articles were assessed for inclusion in this review, with the majority of them accepted. The three exclusion criteria were as follows: (i) studies that were not connected, (ii) papers that were not relevant, and (iii) research that had inadequate data. This resulted in the exclusion of 86, 71, and 24 studies, denoted by the letters E1, E2, and E3, respectively, resulting in a final selection of 231 studies. These studies, which fall under category (i), are studies that are unrelated to one another. These studies either do not include AI or do not demonstrate risk stratification for CVD/stroke in people with ED.

There were 86 studies that were excluded from the selection process, which are represented by the letter E1 in the PRISMA model. Non-relevant studies are the only ones that do not fall under the umbrella term of ED, CVD, and stroke. They are not concentrating their efforts on the ED–CVD–stroke area. In this study, we are solely interested in studies that discuss the relationship between ED and cardiovascular disease and stroke. (ii) If studies demonstrate a link between ED and diabetes, we will not consider it. There were 71 studies in this category, which is represented by the letter E2 in the PRISMA model. These studies with insufficient data were those that did not provide enough information to be included in our analysis because they did not provide enough information. These studies found no evidence of a relationship between ED and CVD or ED and stroke. There were no attempts to have such conversations. There was no consideration given to the relationship between ED and CVD risk factors, such as LBBM. Furthermore, they did not have adequate AI, CVD, or stroke features from which to choose for analysis, as previously stated. (iii) These AI characteristics may be utilized in the development of an architecture for risk stratification in CVD and stroke. These AI features might be single deep learning (DL) models, hybrid deep learning (HDL) models, or neural network parameters for CVD and stroke risk stratification. We discovered 24 studies with inadequate datasets, represented by the letter E3 in the PRISMA model.

## 3. Erectile Dysfunction and Cardiovascular Disease Links: Clinical Evidence

The definition of a health risk is “a characteristic or incident that is associated with a higher probability of a certain result, such as the occurrence of a disease.” [54]. The Framingham Heart Study is a major milestone in terms of identifying risk factors for CVD. The FHS’s work has considerably helped preventive medicine. As a result, the focus shifted from treatment to prevention and education [55]. All combined atherosclerotic plaque risk factors should be considered relevant to CVD [56]. Age, gender, a family background of CVD, and ethnicity should be considered as non-modifiable CVD risk factors. Age is an indicator of duration, and it is linked to CVD risk. Age is also the largest indicator affecting cardiovascular outcomes [57,58]. Another well-known CVD risk factor is the male gender. According to the FHS data, women’s CVD mortality is equivalent to that of males 10 years younger [59]. Another well-established, non-modifiable risk factor is a first-degree relative with a history of CVD [60,61]. This link is especially robust in younger people who have a strong family history of premature illness [62,63]. Even though these risk variables are non-modifiable, their identification is important in clinical treatment because it helps in identifying individuals who require more stringent control of modifiable CVD risk factors.

In addition to ischemic heart disease, stroke, and peripheral artery disease, hypertension has been linked to several of the most significant atherosclerotic symptoms, including peripheral artery disease (PAD) [64,65]. In the normal BP range (>115/75 mmHg), there is no solid evidence of a risk threshold for CVD [65]. This link has been seen in people of all ages, and it appears to be greater for systolic BP than diastolic BP [66,67]. Stroke and heart disease fatalities increase more than multiple times for those aged 40–69 years who have an increase in their blood pressure of 20 or 10 mm Hg [65].

Diabetes mellitus (DM) doubles or triples the risk of myocardial infarction or stroke, as well as the risk of CVD mortality [68,69]. This risk rises in proportion to the degree of glycemic change [70]. Intermediate carbohydrate metabolic anomalies have also been linked to a higher CVD risk [71,72]. In contrast to diabetes, diabetic people have a higher risk of CVD due to the existence of additional metabolic abnormalities [73].

**Figure 3:**
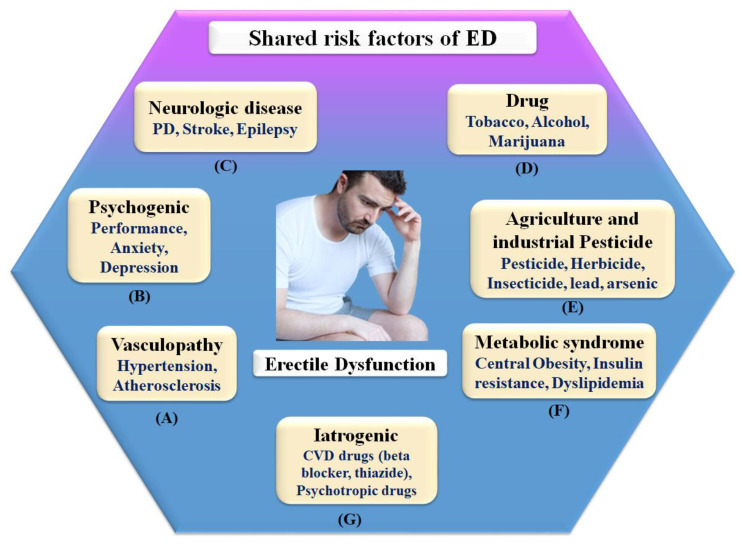
Shared risk factors of ED.

ED is generally referred to as a vascular disease, and it is generally known that it shares several health risks with CVD, including obesity [32,74], chronic renal disease [75], poor socioeconomic status [58], low fruit and vegetable consumption [76], inadequate physical activity [77], metabolic syndrome [78,79], and elevated C-reactive protein levels [80], which are all well-known risk factors for CVD. In this context, a large prospective study evaluating the effect of CVD risk variables on ED over 25 years showed that age, BMI, cholesterol, and triglycerides were all highly associated with ED [79]. Smoking, BMI, hypertension, cholesterol dietary consumption, and unsaturated fat intake have all been linked to an increased risk of ED [78,81]. Figure 3 indicates the shared risk factors of ED.

Therefore, in connection, ED affects around 75% of diabetes patients over the age of 60 and grows proportionately with the severity of the condition [82]. It is possible that ED and penile atherosclerosis are the common denominators between ED and diabetes [83]. However, the link between these two clinical diseases is complex, and additional pathophysiologic processes, such as autonomic neuropathy and hormonal abnormalities, may be involved in the development of these two clinical conditions [22,84].

### 3.1. The Pathophysiologic Link between ED and CVD

The pathophysiology of ED is dependent on the integrity of the endothelium [85,86]. Sexual drive induces the production of NO and other endothelial mediators, resulting in stimulating sympathetic stimulation in the veins feeding penile regions and an enhanced blood flow to the penis while blocking the vein discharge [86,87]. These occurrences cause blood to be trapped within the corpora cavernosa. This increase leads to system pressure and an erection [88]. The carotid arteries hypothesized that ED and COAD have the same involvement in the pathogenesis pathway [89]. ED and circulation stenosis may result from exposure to known risk factors. Due to the systematic character of atherosclerosis, all arterial pathways may be harmed to the same amount, but the onset of signs is linked to arterial size [9,90]. Increased vascular tolerance for the same amount of endothelial dysfunction and/or atherosclerotic burden is observed in bigger vessels when compared to smaller arteries [91]. Alongside the more compact ones, penile veins are smaller than other veins in the body [92]. Compared to coronary arteries, they are tiny, (1–2 mm) to (3–4 mm), with endothelial dysfunction at the very same level, and atherosclerosis may cause a greater decline in blood flow [9].

**Figure 4:**
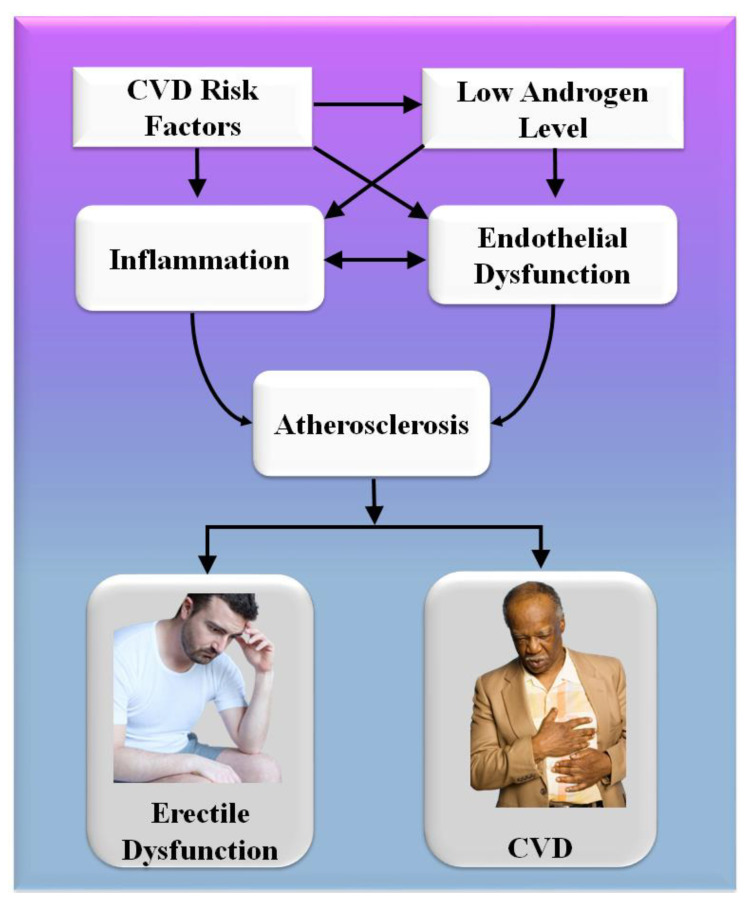
CVD risk factors are linked with inflammation, androgen, and endothelial dysfunction.

Consequently, the vascular system of the penile organ may serve as an early warning system for a wide range of vascular conditions [93]. Individuals with chronic coronary syndromes (CCS) are more likely to have ED than those without CCS, according to this hypothesis. In this respect, Montorsi et al. [3] explained that for patients with chronic coronary syndrome, ED is common before CAD symptoms appear. Most patients with CCS begin to have sexual dysfunction three years before any cardiac symptoms appear. This contrasts with the rarity of sexual dysfunction in those suffering from acute coronary syndrome [3]. Appropriate arterial penile lesions were found in only 12.9% of the cases, compared to a high frequency of 87% in the coronary system and 77% in the internal iliac artery area [94]. Figure 4 shows the CVD risk factors linked with inflammation, androgen, and endothelial dysfunction.

A comprehensive reformulation of all available evidence revealed that, while the artery-size theory is crucial to understanding the complicated relationship between ED and COAD, vasculogenic ED is also connected with dynamic, macroscopically intangible irregularities linked to endothelial dysfunction and neurogenic hyperactivity [42]. The usual indications of cardiovascular problems are quite often disguised in diabetics, causing a diagnostic lag of COAD and difficulty in altering the disease’s natural history [95]. In diabetes patients, an individual relationship between ED and asymptomatic COAD has indeed been described [96,97]. Endothelial functioning is affected by low-grade inflammatory cytokines, which can lead to a thrombogenic state [98]. Several studies have linked the development and intensity of ED to the elevated expression of inflammation biomarkers [44,45,46,47]. The major targets for androgen actions inside the penile and cardiovascular pathways are endothelium and sleek cells, and congenital hypothyroidism is associated with an increased risk of arteriosclerotic remodeling [99,100].

As a result, it is found that people who have ED and risk factors for cardiovascular disease are more likely to have a “silent COAD.” They should get a full CVD examination.

#### Mechanism of Penile Erection

**Figure 5:**
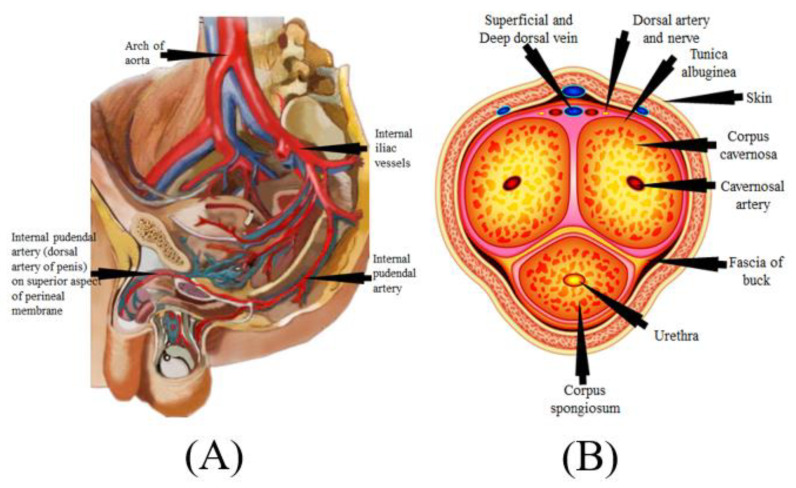
(**A**) Mechanics of penile erection (courtesy of Atheropoint^TM^, Roseville, CA, USA). (**B**) Cross-sectional of the penis (courtesy of Atheropoint^TM^, Roseville, CA, USA).

The mechanism of the male penile erection, as well as cross-section, is shown in Figure 5A,B, where the aorta is directly connected to the penal artery. A significant blood input is essential for successful sexual performance [101,102]. As previously stated, normal penile erection is a neurovascular event that causes sexual stimulation and the release of NO hormones from endothelial cells [103,104].

As a result, strong blood flow from the heart to the penal muscle cells is required for a proper erection [105,106]. All these processes cause blood to be caught inside the corpora cavernosa (Figure 5B), resulting in intracavernous pressure and an erection [107].

### 3.2. The Effect of SARS-CoV-19 on Erectile Dysfunction

**Figure 6:**
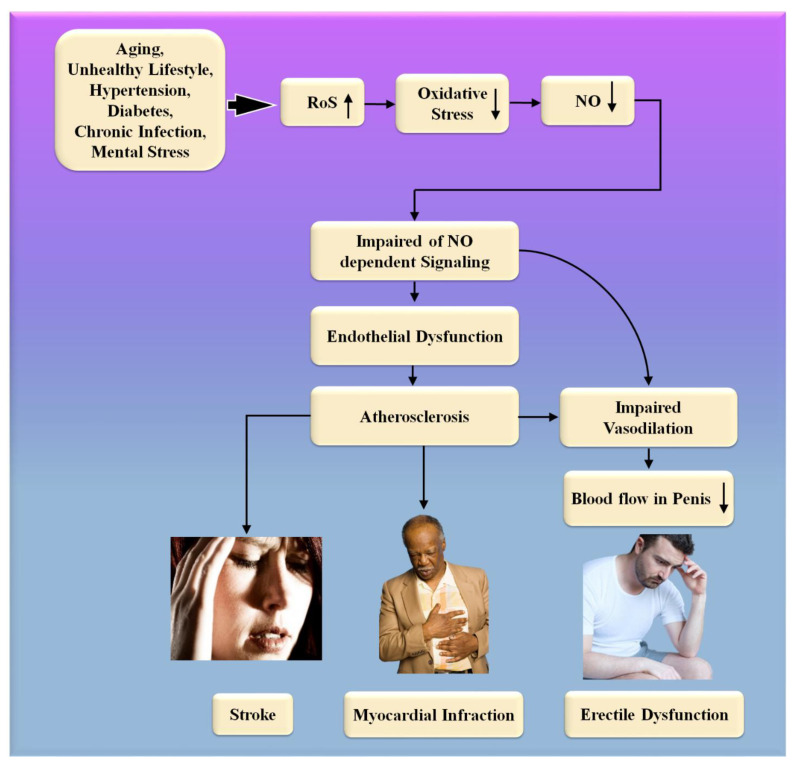
The biological link between ED and CVD/Stroke. RoS: reactive oxides stress, NO: nitric oxide, Up Arrow: depicts increase, Down Arrow: depicts decrease.

**Figure 7:**
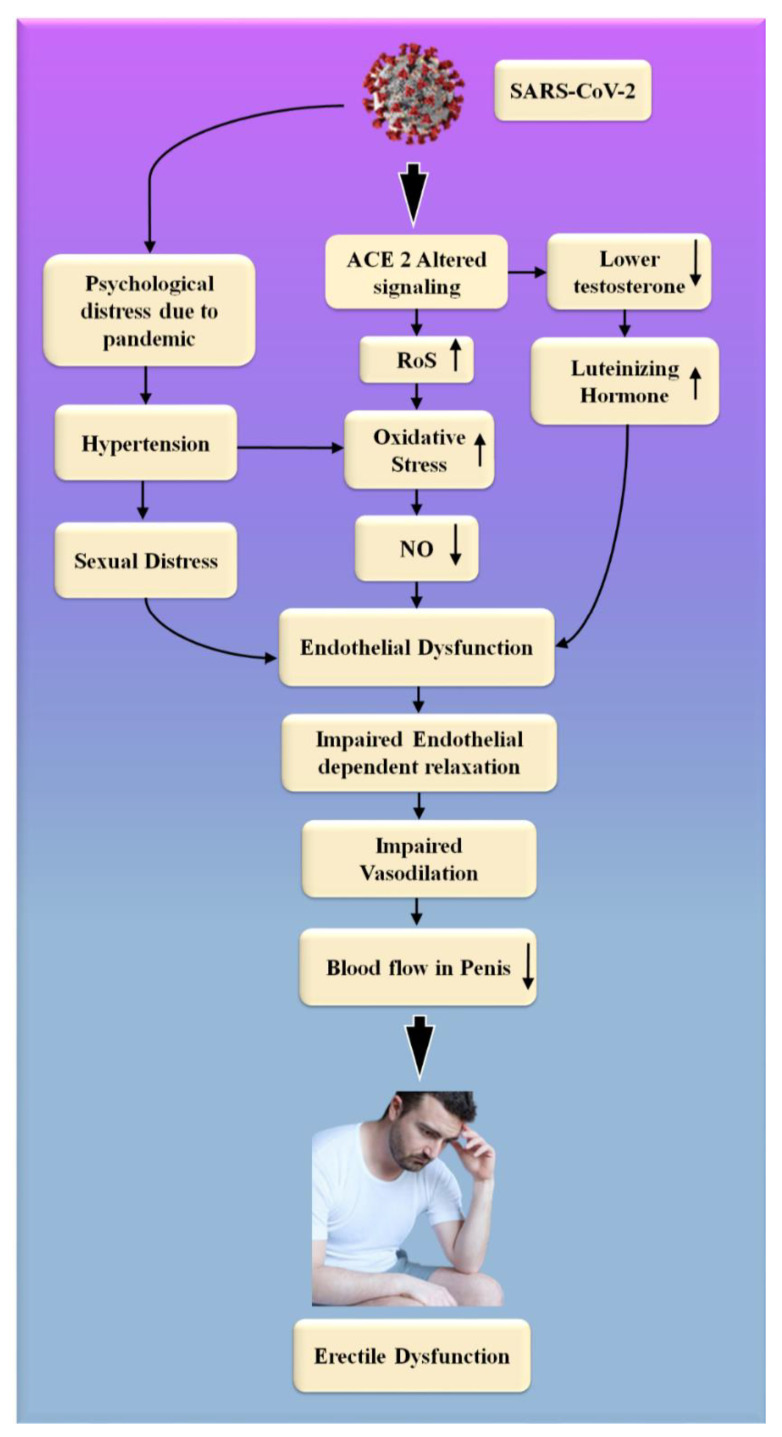
The biological link between COVID-19 and ED. RoS: reactive oxides stress, NO: nitric oxide, Up Arrow: depicts increase, Down Arrow: depicts decrease.

SARS-CoV-2, the interaction of the enhanced ACE2 and the transmembrane protease serine 2 with a component of the spike protein, accelerates binding and transit into vascular endothelium cells [108]. According to the studies, endothelial dysfunction is a significant contributor to COVID-19 symptoms [109,110]. The Table 1 show the relationship between ED with CVD or coronary artery disease. Direct viral invasion of testicular tissue via ACE2 receptors, temperature-related testicular injury resulting from sustained high fever, inflammatory and autoimmune responses, and viral infection-related oxidative stress are some of the suggested causes of this damage [111,112]. Figure 6 explains the biological link between ED and CVD/Stroke and Figure 7 validates the biological link between SARS-CoV-19 with ED.

Endothelial cells infected with SARS-CoV-2 suffer endothelial damage, which causes thromboembolic vascular lumen alteration in the endothelium, immune thrombosis, and reversal in many organs [123]. These are the ultimate and noticeable consequences of the cells taken by SARS-CoV-2 from the endothelium [124]. ED is one of the most common symptoms of COVID-19, which is caused by endothelial dysfunction [123]. This can result in circulatory problems in numerous organs [109,110]. This includes a reduction in blood supply to the testicles, which can lead to ED. Natural nitric oxide (NO), generated by healthy endothelial cells, is an essential cofactor in the endothelium-dependent phase transition in the corpora cavernosa [125]. Endothelial dysfunction is caused by a decrease in eNOS expression, which results in a decrease in NO production [126,127]. Increased endothelium-bound cavernosal tissue vasodilation is associated with hypertension and diabetes [128].

People were experiencing psychological trauma, as well as the overall feeling of a high degree of uncertainty associated with the COVID-19 global epidemic [129]. The restrictive measures that were implemented during this critical period, in the long term, influenced interpersonal and intimate relationships [130]. Concerns about safe intimate/sexual interplay, the forced separation of intimate partners, the escalation of marital disputes, and degradation in contact are some of the most significant contributors to a person’s experience of sexual troubles and sexual unhappiness at this age [130,131,132].

Sexual desire and expression differences, as well as a lack of privacy while confined, have both been linked to the development of sexual difficulties and dissatisfaction [133,134]. COVID-19 infection, on the other hand, has the potential to negatively impact male sexual function by inducing endothelial damage, which can result in erectile dysfunction, testicular injury, and psychological alterations [134,135].

We hypothesized that erectile dysfunction occurs more frequently in the presence of heart issues when the endothelium and smooth muscle are dysfunctional. Endothelial dysfunction impairs blood flow to the heart and the penis, contributing to the development of atherosclerosis.

## 4. Artificial Intelligence-Based System for CVD/Stroke Risk Assessment in ED Patients

Machine learning is a powerful framework because it uses a knowledge-based model to create a training system. Several ML-based applications have been developed in healthcare, spanning subfields of medicine, such as diabetes [38,136,137], neonatology [138], gene analysis [139,140], COAD risk stratification [141,142], EEG-based signal classification [143,144], and CTAD symptomatic vs. asymptomatic plaque classification [145,146,147]. When it comes to risk stratification, ML-based strategies have also dominated cancer imaging paradigms, such as thyroid [148,149,150], breast [151], ovarian [41,152], prostate [153], liver [154,155], and other forms of cancer, such as skin [148,149,150,156,157].

The ability of ML to adjust the non-linearity between a set of risk factors (or covariates) and the gold standard is the second major benefit of ML. Such evidence in the context of CVD risk assessment has recently been introduced [28,158,159,160,161]. These risk factors include (i) conventional office-based, (ii) laboratory-based covariates, and (iii) current drug consumption, while the gold standard criteria are heart failure or stroke. In the CVD/stroke risk paradigm, the inclusion of ED covariates can add value to the CVD risk stratification in ED patients.

### 4.1. Machine and Deep Learning Framework for CVD Risk Assessment in ED Patients

**Figure 8:**
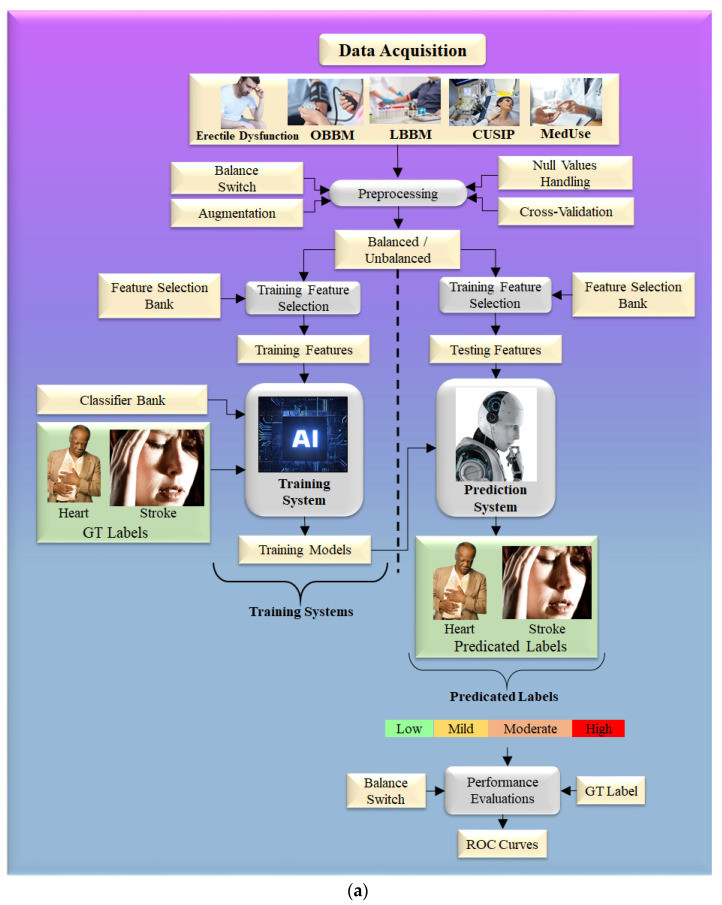

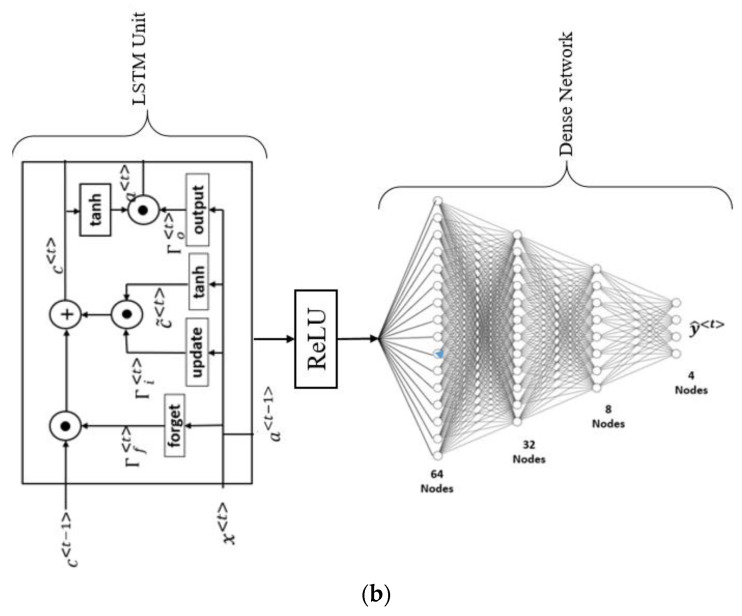
(**a**). Machine learning model to predict the severity of CVD and stroke in ED framework. (**b**). The general structure of LSTM architecture.

The typical variables included the combination of OBBM, LBBM, CUSIP, and MedUSE [67]. For cost reasons, non-invasive protocols for carotid arteries [162] under minimal noise conditions such as harmonic and compound imaging [47,163] are favored for atherosclerosis imaging. The identification of plaque build-up is aided by automated carotid far-wall segmentation [164,165]. Figure 8a shows the step-by-step approach for risk stratification for CVD and stroke risk stratification in ED patients using the AI framework [166]. On the left-hand side is shown the extraction of features using the training dataset, which is then used for model generation using the conventional classifier, given the gold standard. On the right-hand side of Figure 8a is shown the CVD/stroke risk prediction by transforming the testing features based on the training model. Because the input gold standard consists of multiple risk classes of coronary artery disease, the predicted CVD/stroke risk will also be a CVD/stroke granular risk.

One can also use deep learning strategies such as LSTM for CVD risk assessment. The main feature of LSTM is the ability to process multiple types of data points, such as a single (image). The main component of the LSTM architecture is a cell, an update gate, an output gate, and a forget gate (Figure 8b). During random intervals, the cell stores the values, and the three gates control the flow of information or features into and out of the cell [167]. LSTM took the place of the recurrent neural network (RNN), which can address the limitation of the RNN (i.e., simple RNN associated with TensorFlow). LSTM is better at formulating long-term dependencies in the data [168]. The LSTM architecture is displayed in Figure 8b, where the LSMT unit has four fully connected dense layers stacked together. The structural configuration of LSTM is similar to an RNN and well suits for CVD risk stratification in ED patients [169,170]. Even though ML is a powerful paradigm, it requires the features to be manually optimized, unlike in DL, where the features are automatically optimized.

### 4.2. Participating in Studies for CVD Risk Assessment Using AI

Table 2 and Table 3 show six different independent studies for (a) CVD risk prediction and (b) ED prediction, both using the AI framework. There were several different types of ground truth employed in these CVD risk prediction studies, including death, stroke, CHD, and CVD [171,172]. The risk factors that were used were OBBM, LBBM, and CUSIP derived from carotid US scans which are marked as input covariates (IC) in Table 2 and Table 3. As a result, support vector machines (SVMs) were used for the classification, along with logistic regression, a convolution neural network (CNN), an artificial neural network (ANN), a random forest algorithm (RF), and a principal component analysis (PCA). Table 2 and Table 3 contain more information on the characteristics of this classification technique.

Atherosclerosis is a systemic inflammatory disease. The plaque in the coronary artery mirrors that in the carotid artery, especially in the bulb or bifurcation area [173]. Numerous studies have shown cholesterol, fibrosis, fibrin, and calcium in both coronary and peripheral arteries [141]. Several studies have found a strong link between carotid artery plaque measures and the risk of COAD and CVD [56,174,175].

**Table 2:** Generalized studies for prediction of CVD in AI framework using input covariates.

SN	Citations	IC	DS	GT	FE	TOC	ML vs. DL	ACC	AUC
1	Gorek et al. [176] (1997)	OBBM, LBBM	30	Diagnose ED	NR	CNN	DL	80.79	0.80
2	Kellner et al. [177] (2000)	OBBM, LBBM	100	Diagnose ED	NR	CNN	DL	72.79	NA
3	Glavaš et al. [178] (2015)	OBBM, LBBM	185	Diagnose ED	NR	LR, SVN, ANN	ML	74.40	0.812
4	Chen et al. [179] (2019)	LBBM	5664	Predict ED	NR	LR, ANN, SVM, RF	HDL	76.65	0.817
5	Lingli et al. [180] (2018)	OBBM, LBBM	95	Diagnose ED	DT	SVM	ML	96.7	NR
6	Jang et al. [181] (2019)	OBBM, LBBM	187	ED drugs therapy	NR	ANN	DL	100.00	NR

SN: serial number, IC: input covariates, DS: data size, GT: ground truth, OBBM: office-based biomarker, LBBM: laboratory-base biomarker, FE: feature extraction, TOC: type of classifier, ACC (%): percentage accuracy, US: ultrasound, NR: not reported.

**Table 3:** Studies for ED prediction using the AI framework.

SN	Citations	IC	DS	GT	Classifier	TOC	ML/DL	ACC %	AUC
1	Biswas et al. [182] (2018)	OBBM, LBBM (US)	407	Stroke, Diabetes	NR	CNN	DL	99.61	0.99
2	Jamthikar et al. [158] (2019)	OBBM, LBBM (US)	395	CVD	PCA	RF	ML	95.00	0.80
3	Kandha et al. [183] (2020)	OBBM, LBBM	346	Death	CNN	NB, SVM, KNN, DT	DL	83.33	0.833
4	Jamthikar et al. [160] (2020)	OBBM, LBBM, CUSIP	202	CVD	SVM	LR, SVN, ANN	ML	92.53	0.92
5	Saba et al. [184] (2020)	OBBM, LBBM, CUSIP	246	Death	6 Models	SVM	HDL	89.00	0.898

SN: serial number, IC: input covariates, DS: data size, GT: ground truth, OBBM: office-based biomarker, LBBM: laboratory-based biomarker, FE: feature extraction, TOC: type of classifier, ACC: percentage accuracy, US: ultrasound, NR: not reported.

**Figure 9:**
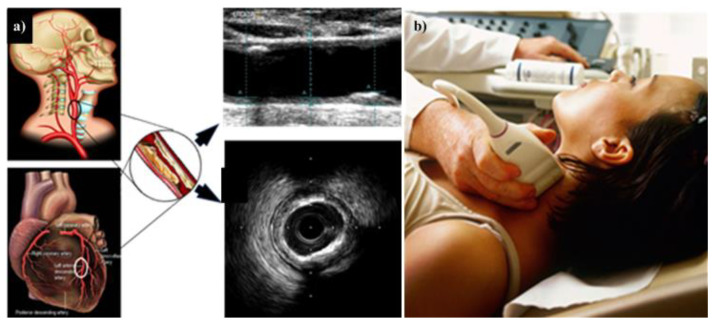
(**a**) The CTAD is being investigated as a potential surrogate marker for COAD. (**b**) Imaging device where the carotid artery is being scanned with the linear ultrasound probe. The middle shows the B-mode carotid longitudinal US scan and IVUS-based coronary artery cross-sectional scan (produced with permission by Atheropoint^TM^, Roseville, CA, USA).

Multiple modalities have been used for imaging the carotid artery. A US is considered more user-friendly, convenient, and cost-effective than an MRI [185]. Figure 9a,b show how the carotid B-mode ultrasound acquisition system can be applied to ED patients [186].

The Carotid IMT and carotid plaque area have been shown in clinical trials [187] to be effective surrogate measures for coronary vascular disease. Additionally, in [188], the authors employed the cIMT on the carotid and coronary arteries in concert with an ultrasound framework. The authors in [189] demonstrated the maximum plaque height (MPH) as a risk factor for COAD. Additionally, the authors in [190,191] demonstrated how the carotid bulb may be used to estimate the risk of COAD.

In a comprehensive risk assessment, we need to be able to automatically and precisely quantify the CUSIP consisting of carotid intima-media thickness, ave., max., and min (cIMTave, cIMTmax, cIMTmin), carotid intima-media thickness variability (cIMTV), morphological total plaque area (mTPA), geometric total plaque area (gTPA), lumen diameter (LD), inter-adventitia diameter (IAD) [184], and composite risk score (CRS) [192]. We require a risk assessment system that can determine the severity of COAD in ED patients. All ED investigations found an increase in cardiovascular illness, which is linked to an increase in phenotypes, such as cIMT, gTPA, mTPA, and CRS [184]. This CUSIP is then used as a covariate in the ML system (Figure 9).

### 4.3. Plaque Tissue Characterization Using Machine Learning/Deep Learning Paradigms

The presence of bad LDL deposits in the bulb over time due to ED raises plaque load, generating wall sheer stress (WSS) in the artery walls, which can lead to plaque rupture [193,194]. Low-intensity asymptomatic plaques are difficult to detect and can rupture, resulting in death [195,196]. However, they cannot be seen with bare eyes, so we need to find a technique that can characterize the plaque. Bright plaques are simple to detect and identify, although calcium deposits can be deceiving [197,198]. It is quite difficult for ultrasound technicians or radiologists to make rapid judgments on plaque lesion characterization due to the time constraint [199,200]. As a result, there is a strong relationship between COAD and CTAD, and it is simple to obtain image phenotypes using a low-cost, non-invasive B-mode carotid longitudinal US scan.

Endothelium, the inner connecting of the arterial wall, and smooth muscle cells are damaged by ED, resulting in damage to the arterial walls of the coronary artery, causing cardiovascular problems [201]. As a result, normal plaque becomes vulnerable or dangerous plaque over time [202]. Due to this, ED can be an important indicator for symptomatic plaque. Furthermore, plaque growth is a multi-focal illness [203]. It does not occur at a single location in space. As a result, the illness spreads intermittently all over the artery’s sidewalls [204]. ML has been used to identify symptomatic plaque for stroke risk stratification, labeled as Atheromatic^TM^ 1.0 (AtheroPoint LLC, Roseville, CA, USA) [157,182].

#### 4.3.1. PTC Using Machine Learning

To identify the severity of CVD risk in mild ED vs. severe ED patients, ML and DL methodologies for carotid plaque tissue characterization (PTC) approaches are required [182,205]. In the clinical imaging area, popular classifiers such as random forest (RF), support vector machine (SVM), decision tree (DT), and AdaBoost have been commonly implemented. The PTC can serve diagnostic and therapeutic requirements while cutting costs because of advancements in US technology. Saba et al. [206] utilized a polling-based PCA approach in an ML framework to choose dominating characteristics for better performance. International cardiologists mostly use ML for CHD risk stratification before stenting and percutaneous coronary intervention treatments [207]. For CVD risk assessment, this study used a technique that combined intravascular ultrasonography (IVUS) greyscale plaque morphology and cIMT.

**Figure 10:**
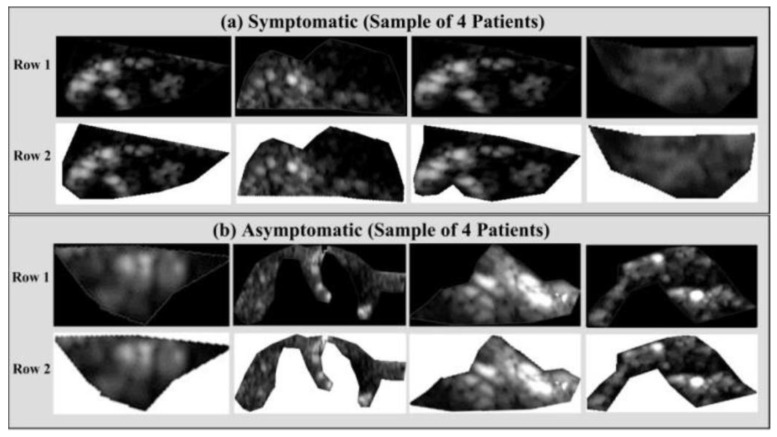
Delineated plaque in the B-mode US. (**a**) Symptomatic plaque and (**b**) asymptomatic plaque (produced with permission by Atheropoint^TM^, Roseville, CA, USA).

Vascular radiologists can promptly diagnose a patient by using the automated characterization of the symptomatic and asymptomatic plaque from US pictures. Acharya et al. [47] used 346 images of US plaques, and out of that, 196 were symptomatic and 150 asymptomatic. Figure 10a,b illustrate two instances of symptomatic (a) and asymptomatic plaque (b). To extract the features, the photos were pre-processed to eliminate noise, and discrete wavelet transform (DWT) was used.

A variety of studies have been conducted in the ML framework to predict the risk of CTAD and COAD [147,184]. Additionally, ML was used to identify individuals with COAD by analyzing the greyscale characteristics of left ventricular ultrasound data [208]. Recently, a deep learning-based technique for predicting the risk of COAD was developed utilizing the carotid artery as a gold standard [183,209,210].

#### 4.3.2. PTC Using Deep Learning

**Figure 11:**
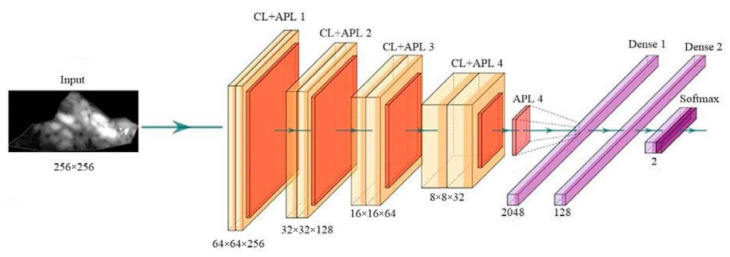
The general structure of CNN architecture (produced with permission by AtheroPoint^TM^, Roseville, CA, USA) [211,212].

With the help of deep learning, PTC can also be used to predict stroke risk. This strategy can be used to predict coronary risk if the gold standard is taken from the coronary artery. Figure 11 shows a convolution neural network-based deep learning used for enhancing the features or extracting useful information from the input of either images or signals. The feature extraction can be performed in two forms, namely 1D or 2D. The main characteristics of the CNN technology are max pooling, convolution, non-linearity, and classification [211].

A thorough review of various studies reveals that ED patients have a higher risk of CVD. Our observations on the hypothesis showed that “ED has a relationship with CVD/stroke and holds via the vascular atherosclerotic pathway”. We further investigated such a setup in the COVID-19 paradigm. As a result, a low-cost B-mode carotid longitudinal US scan could be used for CVD screening in ED patients to prevent the CVD symptoms from worsening to a cardiovascular event or cerebrovascular event.

#### 4.3.3. Recommendations for ED Patients

With the help of an AI-based non-invasive model, these patients may be successfully monitored, and long-term CVD effects can be prevented. We showed how ML and DL can be integrated for CVD/stroke risk stratification with better sensitivity and specificity for ED patients. Such a strategy will improve better statin control for monitoring the CVD/stroke risk. This can be further customized and personalized for individual patients, which is unique and valuable in today’s healthcare systems. This AI model may be used by physicians to advise ED patients by giving further information on CVD and stroke risk.

#### 4.3.4. Manifestation

ED treatment has changed dramatically since the discovery of sildenafil, a phosphodiesterase type 5 inhibitor, which has enabled many more men to seek assistance [213,214]. Three- and five-cyclic guanosine monophosphate, a second messenger for the relaxing effects of nitric oxide on smooth muscle, is inhibited by phosphodiesterase type 5 inhibitors, which have been shown to be effective in clinical trials [215,216]. As a result of sexual stimulation, endothelial cells and nonadrenergic, noncholinergic neurons release NO, which aids in the relaxation of the trabecular erectile tissues as well as dilation of the helicine artery of the penis by increasing the formation of cyclic guanosine monophosphate [217,218]. In response to the increased blood flow, the sinusoidal gaps of the corpora cavernosa grow swollen and suffocate with blood. Because of the engorgement of the tunica albuginea, the subtunical venules that drain the corpora are compressed, resulting in a decreased venous outflow from the penis [219]. As a result, the penile blood pressure rises, leading to the development of a physiological erection.

As an oral erectile dysfunction medication, Tadalafil is a potent selective phosphodiesterase type 5 inhibitor that is currently being researched and developed [217]. Back discomfort, nasal congestion, myalgia, and flushing are among the most commonly reported treatment-related adverse effects of tadalafil. When taken as needed before sexual activity and with no restrictions on food or drink, the pharmaceutical tadalafil significantly improved erectile function, according to the study. It was successful in restoring normal erection function to a large number of people [220].

## 5. Critical Discussions

### 5.1. Principal Findings

We found that ED occurs more frequently in the presence of heart issues when the endothelium and smooth muscle are dysfunctional. Endothelial dysfunction impairs blood flow to the heart and the penis, contributing to the development of atherosclerosis. SARS-CoV-2-infected endothelial cells suffer endothelial damage that results in thromboembolic vascular lumen modification in the endothelium. Moreover, we obtain strong evidence that smoking, BMI, hypertension, cholesterol dietary consumption, and unsaturated fat intake have all been linked to an increased risk of ED. It is feasible to use an AI-based system for the CVD and stroke risk stratification to find the severity of heart failure and stroke in ED patients.

### 5.2. Benchmarking

Following the analysis of various studies, we discovered a few research studies that examined the link between ED with CVD utilizing OBBM, LBBM, and MedUSE. Only a few papers discuss the significance of AI in the diagnosis of CVD and ED independently. Despite the proposed study, no other study uses the AI model to describe the severity of CVD in the ED framework. Table 4 shows the benchmarking analysis of several studies.

Bonetti et al. [113] explained the role of ED as a systemic disorder that plays an important role in the progression of atherosclerosis and its consequences. Growing data reveal that endothelial function is not only determined by the properties of currently recognized cardiovascular risk factors. Endothelial integrity, on the other hand, is based on the balance of all cardiovascular risk factors and vasculoprotective aspects in a specific person, including unknown variables and hereditary susceptibility. Endothelial dysfunction can be used as a predictor of an individual’s atherosclerosis risk. In support of this idea, endothelial dysfunction in the coronary or peripheral circulation has been proven to be a lone indicator of a poor cardiovascular outcome, offering predictive information beyond that obtained through traditional risk factor evaluation.

Montorsi et al. [9] focused on vascular illnesses where ED is a concern caused by COAD, high blood pressure, cerebrovascular disease, peripheral arterial disease, and type 2 diabetes Notably, ED is also common in vascular syndromes, such as COAD, hypertension, cerebrovascular disease, PAD, and diabetes mellitus (DM).). Endothelial dysfunction and late obstructive alterations in the vascular system have been found in patients with ED and other cardiovascular diseases. To explain the connection between ED and CTAD, researchers recently proposed the artery-size hypothesis. Because atherosclerosis is a long-term condition, the damage to the major artery beds should have been uniform.

Diaconu et al. [115] described ED as a symptom of vascular disease that is still in its early stages. ED and CVD are both symptoms of the same illness. ED symptoms often present three to five years earlier than indications of COAD and may serve as a warning sign that CVD is on the way. As a result, male patients with cardiovascular risk factors should be examined for ED regularly. In patients with ED, an aggressive treatment strategy targeting the primary cardiovascular risk factors is indicated to avoid CVD complications and improve their prognosis. Gandaglia et al. [82] showed that the systemic relationship of ED with CVD should be treated as such. By the interaction of CVD risk factors, androgens, and chronic inflammation, there is an increase in the formation of atherosclerosis and flow-limiting stenosis. Endothelial dysfunction and autonomic hyperactivity, which are macroscopically undetectable, may help to explain the complicated link between ED and CVD. The diagnosis of ED frequently occurs before the onset of CVD, providing a golden opportunity for risk mitigation. Patients with ED should have a complete cardiologic examination and obtain comprehensive risk factor management, according to procedures devised specifically for them.

Miner et al. described that the responsibility of physicians is stated as the requirement to assess every man over the age of 40 for the presence or absence of ED, particularly those men who are asymptomatic for COAD signs or symptoms. It is suggested for CVD risk stratification in all men with vasculogenic ED. Another study by Rava et al. [225] provided the first AI-based algorithms capable of reliably and effectively measuring collateral flow in individuals suffering from androgen insensitivity syndrome. This automated technique for evaluating collateral filling may improve clinical decision-making for selecting reperfusion-eligible patients by speeding up the clinical process, reducing bias, and assisting in clinical decision-making.

Mouridsen et al. [221] showed that the use of non-contrast CT and MRI can help distinguish between ischemic and hemorrhagic strokes, which are difficult to distinguish based on clinical symptoms alone. Although an MRI has better sensitivity in an emergency, hypodensity on a CT and DWT and hyperintensity on an MRI detect irreversibly harmed tissue. To our understanding, no study has provided significant useful insight into CVD/stroke risk stratification in the ED paradigm.

### 5.3. A Short Note on Ultrasonography Examination for the Penile Pathology

Of all the causes of impotence, vasculogenic impotence accounts for more than 30%; therefore, ultrasonography is widely preferred for the assessment of penile pathology [226]. Modern ultrasonic examination is based on high-resolution greyscale imaging, which may be used alone or in conjunction with a color and pulsed-wave Doppler. For the examination of vascular reasons in ED, the use of a pharmaceutical stimulant to achieve an erection is currently the standard. Alprostadil (PGE1) and papaverine are the two most often used intracavernous medicines to cause an erection. When phentolamine is combined with these medicines, the amount of stimulant required is reduced, as is the risk of penile discomfort that is occasionally related to PGE1 usage [25,227,228]. Dynamic color–duplex Doppler ultrasonography has been recently proposed for testing high-dose sildenafil [229]. It has fewer false-positive diagnoses and treatments of vascular leakage, but it is time-consuming and requires confirmation in addition to audiovisual sexual excitement [229]. As a result, penile ultrasonography is recommended for the diagnosis of erectile dysfunction.

### 5.4. A Short Note on Bias in AI Systems

AI systems were introduced as an alternative to conventional CVD risk stratification methods [95,114]. However, AI systems have several challenges, such as a tendency to focus primarily on accuracy while ignoring scientific validation and clinical evaluation [45,49]. The disease severity ratio was determined incorrectly due to a lack of solid ground truth selection, such as CVE, coronary CT score, or angiogram stenosis. It places an abundance of emphasis on AI-system reliability while placing an underabundance of emphasis on AI-system authenticity. It causes a bias in the AI system [49]. It is also worth noting that the database contains specific regional patient features, and as a result, the model may produce an under- or over-estimation of the CVD/stroke results for different ethnicities or comorbidities [230]. Thus, for an improvement in CVD/stroke risk stratification in ED patients, it is vital to detect risk-of-bias (RoB) in AI systems [231] and correct CVD/stroke risk stratification. The performance of the AI-based CVD risk stratification can be further improved significantly by merging components such as mobile, cloud, and e-health infrastructure.

### 5.5. Strengths, Weakness, and Extensions of This Study

By identifying a correlation between ED with CVD and stroke, the overall cardio-urologic healthcare systems can be improved. Treatment is certainly preferable to prevention. Patients can be not only treated but also prevented from developing CVD severity if they are (i) aware of the relationship between ED with CVD stroke/ and (ii) low-cost screening using AI-based algorithms as well. One restriction we perceive is that no solid AI-assisted strategy has been developed for treating ED patients with CVD and stroke as variables, and additional research is needed in this area.

Although, there is no clear hypothesis that an AI system exists to forecast the risk of CVD and stroke risk stratification in ED patients, several AI models tackle the challenge of diagnosing CVD, stroke, and ED disorders individually. The lack of multi-center data on ED with CVD and stroke as comorbidities is also a challenge. With the pandemic, it is vital to think about how the SARS-CoV-2 virus may affect both diseases. More systematic reviews of ED-based RoB with comorbidities, such as the SARS-CoV-2 virus, CVD, and stroke, are expected. In the future, we would like to explore how understanding the function of large data is critical for eliminating bias in AI models.

## 6. Conclusions

In this systematic study, the relevance of CVD and stroke risk stratification in ED patients was explored. We also showed how ED problems might lead to vascular and cerebral strokes. As a consequence, recognizing CVD issues in ED patients is crucial. Carotid artery imaging ultrasound has also been found to be a low-cost, non-invasive alternative to traditional imaging modalities for screening CVD and stroke in ED patients. This low-cost B-mode ultrasonography can also be beneficial for the characterization of plaque tissue in ED patients, allowing for better knowledge of CVD and stroke risk stratification in these individuals. Additionally, we showed that AI-based approaches may accurately predict CVD and stroke risk in ED patients. A realistic AI-based model for CVD and stroke stratification in ED patients was described along with the risk of bias in AI. Finally, we discussed the functions of ED in the COVID-19 paradigm, as well as the significance of AI in this context. The study also presented the ED treatment options.

## Figures and Tables

**Table 1:** The studies show the relationship between ED with CVD or coronary artery disease.

SN	Citations	Relation *	ME	PS	Outcome	Treatment
1	Bonetti et al. [113] (2002)	ED with CVD	LBBM	45	ED is a systemic disease that contributes significantly to the advancement of atherosclerosis and its associated complications. There is a need for direct evidence that therapeutic improvements in endothelial function resulted in decreased CVE rates.	NR
2	Montorsi et al. [9] (2005)	ED with CAD	LBBM	34	Because of the progressive or simultaneous alterations in microvascular and macrovascular function, ED is fundamentally an atherosclerotic disorder in its origin and progression.	NR
3	Kirby et al. [16] (2005)	ED with CAD	OBBM	NR	ED and COAD are two distinct clinical manifestations of the same systemic illness, with pathological causes and risk factors that are quite similar to one another. Because of increased understanding of the emergency department as a barometer for cardiovascular health, it is possible to take early action to reduce future CV risk.	NR
4	Vlachopoulos et al. [114] (2007)	ED with CAD	LBBM	NR	ED, inflammation, and low testosterone levels in the bloodstream are all risk factors and pathophysiological links that are shared by cardiovascular disease and erectile dysfunction.	NR
5	Diaconu et al. [115] (2011)	ED with CVD	OBBM, LBBM	231	Both erectile dysfunction and CVD are symptoms of the same illness. ED symptoms often appear three to five years before the onset of symptoms of coronary artery disease, and they may serve as an early warning indication that CVD is on the verge of manifesting itself. As a result, male patients with CVD risk factors should have their erectile dysfunction checked regularly.	phosphodiesterase-5 inhibitors, alprostadil (prostaglandin E1) intracavernous injections, alternatives for the management of ED.
6	Yannas et al. [54] (2011)	ED with CVD	OBBM, LBBM	NR	ED is a sign of cardiovascular disease. As a result, guys with ED should be thoroughly evaluated for cardiovascular risk factors to avoid future CVE (MACE).	NR
7	Gandaglia et al. [82] (2014)	ED With CVD	LBBM	NR	ED and cardiovascular disease (CVD) are two symptoms of the same systemic illness. Atherosclerosis and blood vessel constriction are caused by the interplay of CV risk factors, androgens, and chronic inflammation in the blood vessels. Endothelial dysfunction and autonomic hyperactivity, for example, are both isotropic alterations in the body.	NR
8	Lim et al. [13] (2018)	ED with CVD	OBBM, LBBM	1757	Distinguishing between symptoms of ED and cardiovascular disease (CVD) demands a distinct strategy. Atherosclerosis and vascular constriction are associated with each other, and this association is generated by the combination of CV risk factors, androgens, and chronic inflammation. Atherosclerosis and autonomic hyperactivity are both apparent alterations that are isotropic.	NR
9	Roushias et al. [116] (2018)	ED with CVD	OBBM, LBBM	1768	Endothelial dysfunction is a common denominator in the pathophysiology of both erectile dysfunction and cardiovascular disease. ED is a warning symptom of endothelial dysfunction and a risk factor for cardiovascular disease. Early detection and assessment of ED redefines the risk of cardiovascular disease and allows for earlier intervention. Patients with cardiovascular disease should be treated and monitored more closely if they develop erectile dysfunction.	NR
10	Miner et al. [117] (2019)	ED with COAD	LBBM	242	Angiographic studies show that ED patients under the age of 60 had more severe COAD. This connection is independent of COAD and ED risk factors.	NR
11	Sayadi et al. [118] (2021)	ED with COAD	OBBM	100	COAD is an indicator of atherosclerosis. As a result, the IIEF questionnaire can help diagnose COAD early on.	NR
12	Kałka et al. [119] (2021)	ED with COAD	OBBM, LBBM	751	Sexual health concerns are crucial in cardiac patients. ED predicts CVD due to shared risk factors and pathophysiology. Hypertension, dyslipidemia, smoking, diabetes, obesity, and a poor diet all contribute to vascular endothelium dysfunction.	NR
13	Inman et al. [120] (2021)	ED with COAD	LBBM	1402	ED and CAD may be signs of the same vascular illness. In young men, ED increases the risk of future cardiac incidents, but in older men, it appears to have little predictive value.	NR
14	Imprialos et al. [121] (2021)	ED with CVD	LBBM	NR	Erectile dysfunction is a major health condition that affects many people, and it is more common in people with cardiovascular risk factors or illnesses. Both ED and CVD share pathophysiological pathways.	Patients with or without cardiovascular illness can use phosphodiesterase type 5 inhibitors as first-line ED treatment.
15	Rinkūnienė et al. [122] (2021)	ED with CVD	LBBM	171	ED is common in guys who have had a MI. Men with a history of MI had greater traditional CVD risk factors. Men with ED who have had a MI are more prone to AH.	NR

* SN: serial number, RELATION: effect of ED on CVD, ME: method of evaluation, PS: patient size, OE: outcome, TE: treatment, NR: not reported, MI: myocardial interaction, OBBM: office-based biomarker, LBBM: lab-based biomarker, NR: not reported.

**Table 4:** Comparative analysis of studies with CVD and stroke risk stratification in ED patients.

SN	Citations	Year	Covariates	CVD	Stroke	ED	AI
1	Bonetti et al. [113]	2002	OBBM, LBBM	✗	✓	✓	✗
2	Montorsi et al. [9]	2005	OBBM, LBBM	✓	✗	✓	✗
3	Diaconu et al. [115]	2011	OBBM, LBBM	✗	✓	✓	✗
4	Gandaglia et al. [82]	2014	OBBM, LBBM	✓	✗	✓	✗
5	Miner et al. [117]	2019	OBBM, LBBM	✗	✓	✓	✗
6	Mouridsen et al. [221]	2020	OBBM, LBBM	✗	✗	✓	✓
7	Jamthikar et al. [30]	2020	OBBM, LBBM	✓	✗	✗	✓
8	Bikias et al. [222]	2021	LBBM	✓	✗	✗	✗
9	Reva et al. [223]	2021	OBBM, LBBM	✓	✗	✗	✓
10	Bermejo et al. [224]	2021	OBBM, LBBM	✓	✓	✗	✗
11	Proposed Study	2022	OBBM, LBBM, CUSIP	✗	✓	✓	✗

ED: erectile dysfunction, CVD: cardiovascular disease, AI: artificial intelligence, OBBM: office-based, LBBM: laboratory-based, CUSIP: carotid ultrasound image phenotype, ✓: yes, ✗: no.

## Data Availability

No data availability.

## Acronym Table

**SN**	**Abbreviation**	**Definition**	**SN**	**Abbreviation**	**Definition**
1	ANS	Autonomic nervous system	34	LBBM	Laboratory-based biomarker
2	ANN	Artificial neural setwork	35	LSTM	Long short-term memory
3	ACE2	Angiotensin Converting Enzyme 2	36	MedUSE	Medication use
4	AUC	Area-under-the-curve	37	ML	Machine learning
5	AI	Artificial intelligence	38	MI	Myocardial infarction
6	BMI	Body mass index	39	MRI	Magnetic resonance imaging
7	BP	Blood pressure	40	MACE	Major adverse cardiac events
8	CTAD	Cortaid artery disease	41	NPV	Negative predictive value
9	COAD	Coronary artery disease	42	NB	Naive byes
10	CAS	Coronary artery syndrome	43	nOH	Neurogenic orthostatic hypotension
11	CPD	Chorionic pulmonary disease	44	NO	Nitric oxide
12	CCS	Chronic coronary syndromes	45	Non-ML	Non-machine learning
13	CKD	Chronic kidney disease	46	NN	Neural networks
14	CT	Computed tomography	47	OBBM	Office-based biomarker
15	CUSIP	Carotid ultrasound image phenotype	48	OH	Orthostatic hypotension
16	CV	Cross-validation	49	PAD	Peripheral arterial disease
17	CVD	Cardiovascular disease	50	PRISMA	Preferred reporting items for systematic reviews and meta-analyses
18	CNN	Convolution neural network	51	PD	Parkinson disease
19	CHD	Congenital heart defects	52	PE	Premature ejaculation
20	CCS	Chronic coronary syndromes	53	PPV	Positive predictive value
21	DL	Deep learning	54	PCA	Principal component analysis
22	DM	Diabetes mellitus	55	pCAD	psoriasis computer-aided diagnosis
23	DT	Decision tree	56	RA	Rheumatoid arthritis
24	EMG	Electromyography	57	RF	Random forest
25	ED	Erectile dysfunction	58	RoB	Risk of bias
26	FHS	Framingham Heart Study	59	ROC	Receiver operating-characteristics
27	GT	Ground truth	60	RoS	Reactive oxygen species
28	HTN	Hypertension	61	RNN	Recurrent neural network
29	HDL	Hybrid deep learning	62	SCORE	Systematic coronary risk evaluation
30	HDLC	High-density lipoprotein cholesterol	63	SMOTE	Synthetic minority over-sampling technique
31	IMT	Intima-media thickness	64	SVM	Support vector machine
32	IHD	Ischaemic heart disease	65	US	Ultrasound
33	LDLC	Low-density lipoprotein cholesterol	66	WSS	Wall shear stress
